# Factors influencing tobacco quitting: findings from National Tobacco-Quitline Services, Mumbai, India

**DOI:** 10.3332/ecancer.2024.1777

**Published:** 2024-09-25

**Authors:** Atul Budukh, Sharyu Mhamane, Sonali Bagal, Priyal Chakravarti, Ganesh Ogale, Radhika Sharma, Manisha Yadav, Sushama Saoba, Suvarna Gore, Pankaj Chaturvedi

**Affiliations:** 1 Centre for Cancer Epidemiology (ACTREC), Tata Memorial Centre, Navi Mumbai 410210, India; 2 Homi Bhabha National Institute, Training School Complex, Anushaktinagar, Mumbai 400094, India; 3 Advanced Centre for Treatment, Research and Education in Cancer (ACTREC), Tata Memorial Centre, Navi Mumbai 410210, India; a https://orcid.org/0000-0001-6723-802X; b https://orcid.org/0000-0002-7406-8134; c https://orcid.org/0000-0002-2510-1751; d https://orcid.org/0000-0003-2163-796X; e https://orcid.org/0009-0002-0610-4292; f https://orcid.org/0009-0007-2155-4220; g https://orcid.org/0009-0002-8774-6739; h https://orcid.org/0000-0002-4922-8327; i https://orcid.org/0000-0002-2866-5480; j https://orcid.org/0000-0002-3520-1342

**Keywords:** India, smokeless tobacco, telephone counselling, tobacco cessation, tobacco use

## Abstract

The Government of India established National Tobacco Quitline Services (NTQLS) to provide free and effective telephonic counselling to help people quit tobacco. The objective of the paper is to present the data of tobacco quitters who quit tobacco through NTQLS, Mumbai, in the years 2021–2022 and the factors that influenced tobacco quitting. This is a prospective study where individuals willing to quit tobacco utilised NTQLS. Effective counselling was provided and was followed up. Multiple logistic regression analysis was conducted. Tobacco quitting is the dependent variable while sociodemographic characteristics, tobacco consumption habits, previous quit attempts, alcohol consumption, other substance use and co-morbidity were independent variables. In the years 2021–2022, a total of 448,893 calls hit the system. Of these, 127,163 (28.3%) calls were attended. Of the attended calls, a quit date was set for 21,504 calls (16.9%); of these, 8,276 (38.5%) callers quit tobacco. Individuals with no previous quit attempts [OR: 1.48, 95% confidence interval (CI): 1.25–1.75], never consumed alcohol (OR: 1.37, 95%CI: 1.2–1.56), consumed tobacco within 6–30 minutes (OR: 1.29, 95% CI: 1.12–1.49) and 30–60 minutes after waking up (OR: 1.26, 95% CI: 1.05–1.51) had higher quitting rates. While, female callers (OR: 0.59, 95% CI: 0.35–0.99), private sector workers (OR: 0.70, 95% CI: 0.61–0.81), individuals consuming more than ten tobacco units/packets (OR: 0.70, 95% CI: 0.61–0.79), tobacco use more than 10 years (OR: 0.85, 95% CI: 0.73–0.97), expenditure of more than 5,000 rupees on tobacco (OR: 0.58, 95% CI: 0.44–0.77) and those with no known co-morbid conditions (OR: 0.8, 95% CI: 0.71–0.91) were less likely to quit tobacco. Reduced tobacco consumption will inadvertently reduce the non-communicable disease (NCD) burden and help in achieving the sustainable development goals related to tobacco control and NCD. Quitline plays an important role in tobacco control.

## Introduction

Tobacco use is a modifiable risk factor for a majority of non-communicable diseases (NCDs) and is considered an epidemic of highest public health concern. Globally, claiming the lives of more than 8 million people every year, tobacco kills half of its users [1]. Out of the 1.3 billion worldwide tobacco consumers, 80% belong to low and middle-income countries (LMICs) [1]. The persistent use of tobacco consumption burdens the economy with additional social, environmental and healthcare costs; derailing the countries from their track to achieve sustainable development goals (SDGs) [2].

Worldwide, tobacco consumption shows a slow and persistent declining trend [3]. According to the Fourth World Health Organisation (WHO) Global Trends Report, Southeast Asia records to have the highest tobacco use as compared to other regions; however, it is also the region with the fastest decline in tobacco use [4]. Through the years 2010–2017, a 6% decline in tobacco use in India has been recorded [5].

In India, according to the Global Adult Tobacco Survey (GATS)-2 (2016–17); 38.5% of the current smokers and 33.2% of the smokeless tobacco users have attempted to quit tobacco in the past 12 months while 55% of smokers and 50% of smokeless tobacco users are willing to quit tobacco [5].

To cater to the tobacco cessation needs of the population, the Ministry of Health and Family Welfare (MoHFW), the Government of India introduced the National Tobacco Quitline Services (NTQLS), in May 2016. The NTQLS provides a toll-free number 1800-11-2356 [6]. Currently, there are four NTQLS centres across India, Vallabhbhai Patel Chest Institute (VPCI), Delhi; Dr. Bhubaneswar Borooah Cancer Institute (BBCI), Guwahati; National Institute of Mental Health and Neuro Sciences, Bengaluru; and Tata Memorial Centre (TMC), Mumbai [7].

**Figure 1.**
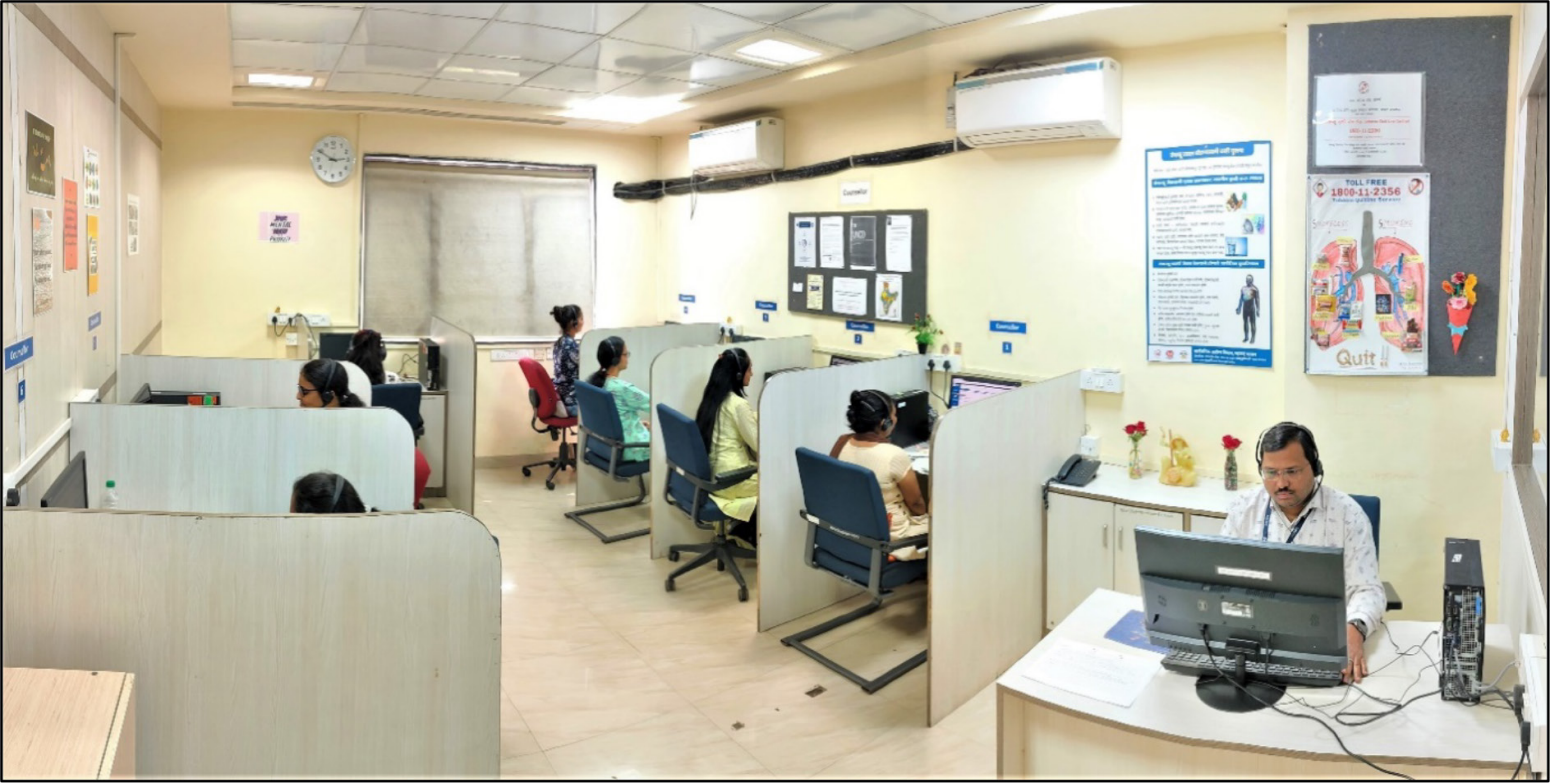
Counsellors attending calls at NTQLS Centre, Mumbai.

**Figure 2.**
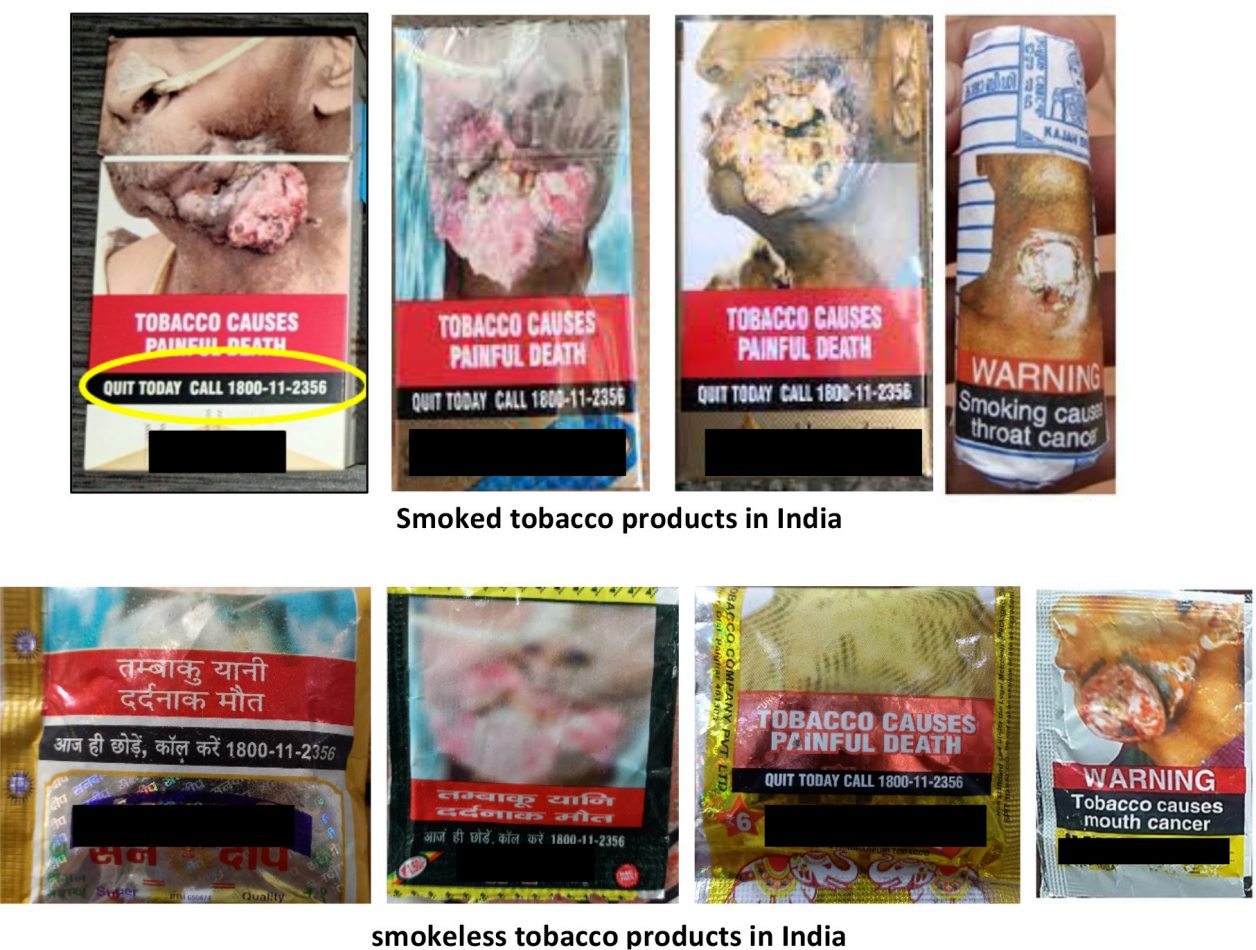
Statutory warning and tobacco quit line toll-free number on smoke and smokeless tobacco products in India.

The NTQLS, Mumbai started in February 2019 [8, 9] at the Centre for Cancer Epidemiology (CCE), Advanced Centre for Treatment, Research and Education in Cancer, TMC, Mumbai, India. The NTQLS, Mumbai provides free tobacco cessation counselling. The services are operational 6 days a week (except Monday) from 8 AM to 8 PM [6]. The working of counsellors attending calls at NTQLS Centre, Mumbai, is shown in Figure 1. Quitline services are proven to increase the chances of quitting and successfully achieving sustained abstinence from tobacco [8]. The person willing to quit tobacco can call the toll-free number. Under the amended Cigarettes and Other Tobacco Products Act (Packaging and Labelling) 2018, it is mandatory and legally binding for the tobacco product manufacturers to print this toll-free number stating ‘QUIT TODAY CALL 1800-11-2356’ with statutory warnings such as ‘TOBACCO CAUSES CANCER’ or ‘TOBACCO CAUSES PAINFUL DEATH’ which is printed on all tobacco products [6]. The statutory warning and tobacco quit line toll-free number on smoke and smokeless tobacco products in India is shown in Figure 2.

There are immediate and long-term benefits to quitting tobacco at any age. An example of the immediate effect of tobacco quitting involves improved lung function, heart rate and drop in blood carbon monoxide level within 2–12 weeks of quitting tobacco, while in a year; the risk of coronary heart disease is reduced to half as compared to tobacco users [10].

Tobacco quitting requires tackling at various levels of behavioural intervention. The NTQLS harnesses the potential of inducing behavioural modification through telephonic counselling to help people quit tobacco. NTQLS provides a service that can be accessed from anywhere, free of cost and provides a feasible option to quit tobacco [9].

However, there are several factors known to influence tobacco quitting. These include a lack of social support, motivation or knowledge about the harmful effects of its use and addiction. These factors may vary with the region and the population concerned [11–15]. Literature from Indian studies to understand the predictors of tobacco cessation gives mixed findings [3, 15–18]. A study by Shaikh and Saikia [3] finds that low-wealth asset scores had higher odds of tobacco quitting while this is opposed to Srivastava’s finding [15] where a significant association of tobacco quitting with socioeconomic status (SES) was seen. A higher quitting was observed among the male gender, higher levels of education and those belonging to the higher wealth quintile. Corsi *et al* [16] state the odds of tobacco quitting were higher in females as compared to males. While Nargis *et al* [17] did not find any significant association between SES and tobacco cessation.

Thus, a thorough understanding of the social, economic and habitué factors is essential to plan and implement interventions [2, 15, 19, 20]. The paper aims to present the data of tobacco quitters who quit tobacco through NTQLS, TMC Mumbai in the year 2021–2022 and factors that influenced tobacco quitting.

## Methodology

### Area covered

The NTQLS, TMC Mumbai is allotted three states; Maharashtra, Gujarat and Goa and three Union territories (UTs); Lakshadweep, Daman and Diu, Dadra and Nagar Haveli. However, it received calls from other Indian states and UTs. According to NTQLS, TMC Mumbai report, in 2021 and 2022, the majority of the quitters were callers from Maharashtra (59.7%) followed by Gujrat (20.4%), Rajasthan (7%) and Uttar Pradesh (4.7%) [21].

### Infrastructure and training

As it is a telephone-based service, a separate telephone primary rate interface line from Mahanagar Telephone Nigam Limited was procured. The telephone was mapped with the national toll-free number 1800-11-2356 and 9 computers were installed with one server. Call centre software was procured and installed on all the computers. Infrastructure has the furniture and other essential items with dedicated office space.

The selected staff was trained by the senior faculty at CCE-TMC for 2 weeks on tobacco hazards, behaviour change and quitline protocol. For acquainting the staff with the quitline working, they were deputed to VPCI, Delhi. Currently, there are 16 counsellors and two supervisors working in two shifts at NTQLS, TMC Mumbai [21]. NTQLS, TMC Mumbai sensitized virtually the Maharashtra state government officials, the public health officers and Primary Health Centre staff regarding the availability of quitline service provided.

The telephonic counselling at NTQLS is provided by trained counsellors using the WHO protocol of telephone counselling. It is a 15–30-minute counselling session and regular follow-up based on 5 A’s (Ask, Advise, Assess, Assist and Arrange) and 5R’s (Relevance, Risks, Rewards, Roadblocks, Repetition) [22]. Quitline services provide tobacco cessation services for both smoked and smokeless tobacco [23]. Regular quality control of the calls of the quitters is performed by the senior supervisor of NTQLS, TMC Mumbai. An independent evaluation of the NTQLS, TMC Mumbai was done by the Post Graduate Institute of Medical Education and Research, Chandigarh, India, through WHO and MoHFW, India [24]. The purpose of the evaluation was to assess the caller characteristics, caller satisfaction, cost-effectiveness of NTQLS and service monitoring of the NTQLS centres in India. The findings of the evaluation reported that NTQLS, Mumbai, was adhering to the standard protocols and service provision was satisfactory [24].

### Call structure and call sequence

The call structure is described in the NTQLS report [9]. The person willing to quit tobacco calls on the toll-free number. The caller is greeted with an automated interactive voice response for language selection through which the system interacts with callers and leads them to the available counsellor.

The first call made by the caller to NTQLS is called the ‘Fresh call’. This call is answered by the counsellor who briefs the caller about the process and the services provided by NTQLS. Since it is a telephone conversation, the counsellor takes verbal consent from the caller and specifies that the data collected will be confidential and will be used for research purposes only. The caller’s details are retrieved and recorded only after his/her verbal consent.

During the ‘Fresh call’, information regarding tobacco use and other sociodemographics of the caller is noted. Based on the caller’s readiness (assessed by the counsellor during the fresh calls to check the willingness of the caller to quit tobacco), a quit date is set by the counsellor. The set quit date is the 7th day after the Fresh call. The caller is guided to quit the tobacco with the help of behavioural modification to deal with the triggers for tobacco consumption, withdrawal symptoms and precautionary measures to prevent relapse. The details of the topics covered during the calls are mentioned elsewhere [9].

The next call is a reminder call or ‘P1 call’, made by the counsellor to the caller 2 days before the set quit date. The counsellor questions the caller regarding the gradual reduction of tobacco use, whether the caller is following the remedies suggested by the counsellor to quit tobacco, triggers, withdrawal symptoms, cravings, hindrances to quit and counsels accordingly, therefore, preparing the caller for the quit date.

The next call is a ‘P2 call’ or ‘Quit date call’ made by the counsellor on the set quit date agreed upon by the caller. Any issues or problems faced by the caller regarding quitting tobacco are discussed. During this call, if the caller confirms quitting tobacco (complete abstinence from all forms of tobacco); only then his/her status of tobacco quitting is considered as ‘Quit’; however, the caller is not yet declared as a ‘Quitter’.

If the caller is unable to quit on the set quit date, an extended quit date is set by the counsellor again and ‘P2 calls’ are made again.

The next call is the ‘P3 call’ which is the follow-up call made after 1 week by the counsellor to those confirmed to quit tobacco during the ‘P2 call’. This is to check whether or not the caller is maintaining his/her ‘Quit’ status confirmed during the P2 call.

The next follow-up call is the ‘P4 call’, which is made 1 week after the P3 call. This is to check whether the caller is continuing to abstain from tobacco. If the caller has successfully stayed quiet since the quit date, only then he/she is declared a quitter during the P4 call.

After this, the follow-up calls from P5 to P8 are made after every 3 months, respectively. The callers continuing to stay quit at P4; that is complete abstinence from all forms of tobacco declared as tobacco quitters.

**Figure 3.**
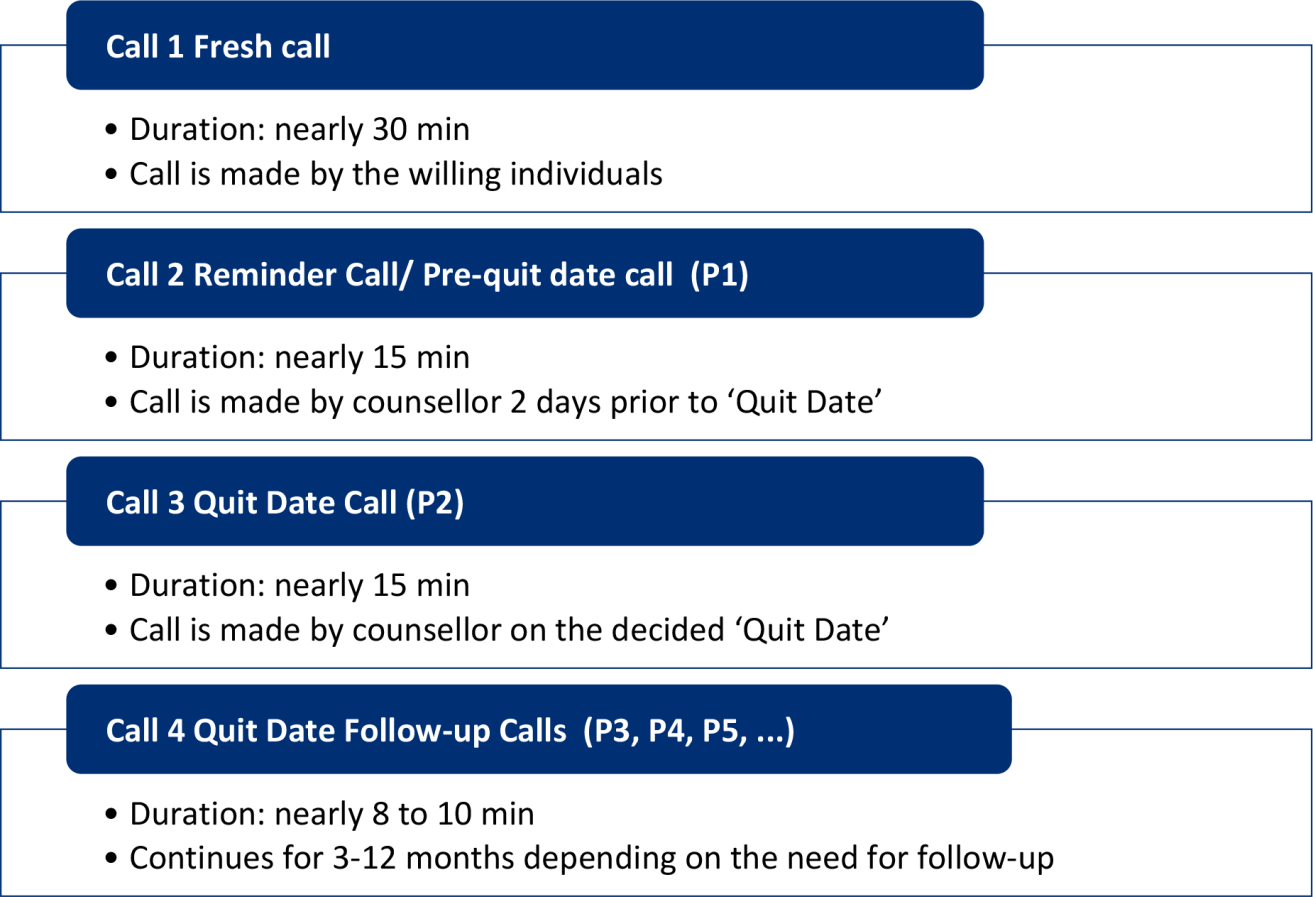
Call sequence at NTQLS centre, Mumbai.

The progress of these calls is recorded on the progress sheet. The follow-up continues for around 6–12 months post-quitting of the caller. Despite the follow-up calls, few callers might relapse and some are lost to follow-up. The callers that relapse at any stage are transferred to P2 calls, where the quit date is set and the quitting journey begins again. The call sequence is illustrated in Figure 3.

### Data collection process and tools used

For the study, we have used the data of the quitters at the P4 stage for the years 2021 and 2022. The data were abstracted from the call centre software into Microsoft Excel. The data were cleaned and checked by the senior staff for validity and further analysis was conducted using STATA version 15. All the data quality measures were implemented.

### Statistical analysis

A univariate analysis was conducted and those variables that were significantly associated were put in a multiple logistic regression analysis to identify potential predictors of tobacco quitting. Tobacco quitting is a dependent variable and the independent variable consists of factors such as the caller’s sociodemographic characteristics, tobacco consumption habits, previous quit attempts, alcohol consumption, other substance use and co-morbidity. The confounders were identified and adjusted during regression. Odds are given in terms of the likelihood of quitting tobacco at a 95% confidence interval (CI).

## Results

**Figure 4.**
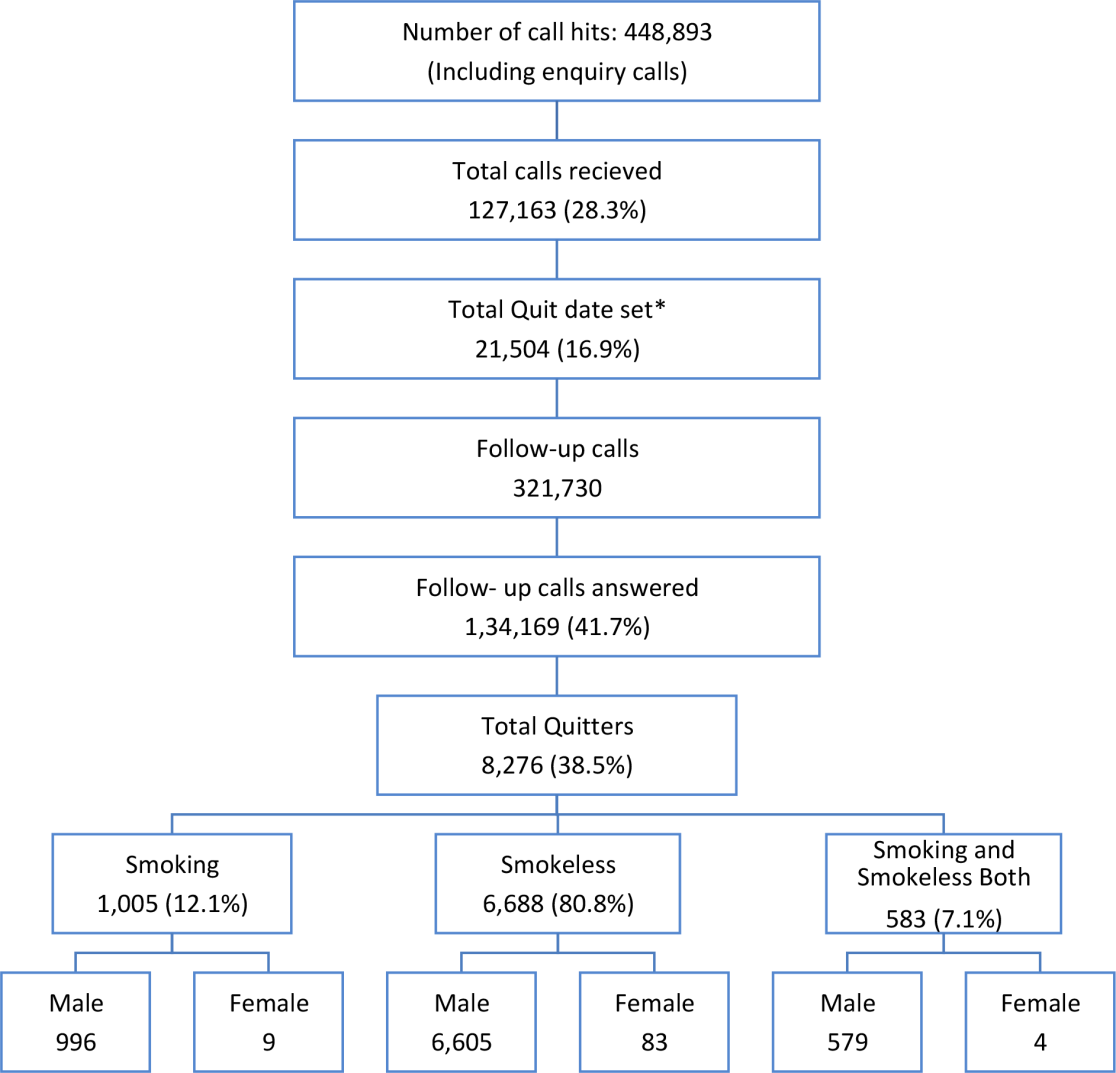
Number of calls received, quit date set and quitters at NTQLS Centre, TMC Mumbai (2021–2022). *Quit date include the ones that were set only in the year 2021–2022 and the previous year’s set quit date is not included.

In the years 2021–2022, the total number of calls that hit the system was 448,893, and out of these, 127,163 (28.3 %) were calls attended. In these years, out of the total attended calls, a quit date was set for 21,504 calls (16.9%), and out of these, 8,276 (38.5%) callers quit tobacco. The details are illustrated in Figure 4. Table 1 illustrates the age distribution of the calls for which the quit date was set. The majority of the calls for which the quit date was set belonged to the 25–34 age group 39.3% while about 1/3rd of it belonged to the age group 18–24.

Out of the total observations of 8,276 tobacco quitters, there was missing information on some of the study variables for 1,641 observations. These 1,641 observations were excluded from the study and statistical analyses were done for the 6,635 common observations of tobacco quitters.

Frequency distribution is presented for 8,276 quitters irrespective of missing information. Table 2 gives the percentage distribution of the quitters and non-quitters for the year 2021–2022. Quitters are the number of tobacco users that quit tobacco in the years 2021–2022, while non-quitters include the ones who could not quit tobacco or relapsed in the year 2021–2022 irrespective of the timeframe in which the quit date was set. For example; non-quitters also include callers for whom the quit date was set in 2020 but found to be relapsed or could not quit during follow-up calls made in the years 2021–2022.

Out of the total 8,276 quitters, 75.6% were aged below 35 and 98.9% quitters were male. Of the total quitters, 35% were drivers/farmers/labourers by profession and 75.7% had a monthly income of less than Indian Rupees (INR) 30,000/-.

For the tobacco consumption habits, 80.8% were smokeless tobacco quitters. A total of 42.4% had a history of using tobacco for up to 10 years, 53.5% used tobacco within 5 minutes of waking up, 43.7% consumed tobacco more than 10 units/packets a day and 74.3% had previous tobacco quit attempts. Of the tobacco quitters, 77.5% did not consume alcohol, 76.6% had no history of substance use and 59% of the quitters had no known co-morbidities.

A multiple logistic regression model was fitted on 6,635 common observations to obtain the factors influencing tobacco quitting. The findings from the multiple logistic regression are illustrated in Table 3.

### Interpretation

#### Gender

Female callers were 41% less likely (OR: 0.59, 95% CI: 0.35–0.99) to quit tobacco as compared to male callers.

#### Occupation

People working in the private sector were 30% less likely (OR: 0.70, 95% CI: 0.61–0.81) to quit tobacco than drivers/farmers/labourers.

#### Duration of tobacco use (In years)

Compared to those who used tobacco for up to 10 years, people who used tobacco for more than 10 years were 15% less likely (OR: 0.85, 95% CI: 0.73–0.97) to give up the habit.

#### Duration of tobacco use on waking up (In minutes)

Individuals who consume tobacco within 6–30 minutes (OR: 1.29, 95% CI: 1.12–1.49) and 30–60 minutes after waking up (OR: 1.26, 95% CI: 1.05–1.51) in the morning have a 29% and 26% higher quitting rate, respectively, as compared to those who were consuming it within 5 minutes of waking.

#### Number of tobacco units (smoked) or packets (smokeless) consumed per day

Those individuals who consumed more than 10 units/packets of tobacco per day were 30% less likely (OR: 0.70, 95% CI: 0.61–0.79) to quit than those who consumed less than ten tobacco pieces per day.

#### Previous quit attempt

Individuals with no previous tobacco quit attempt had a 48% (OR: 1.48, 95% CI: 1.25–1.75) higher quit rate than those attempting to quit tobacco at least once.

#### Monthly expenditure on tobacco

Callers who were spending more than INR 5,000 per month on tobacco were 42% (OR: 0.58, 95% CI: 0.44–0.77) less likely to quit tobacco than those who were spending less than INR 1,000 per month on tobacco.

#### Alcohol consumption

As compared to the individuals who consumed alcohol, the individuals who never consumed alcohol were 37% more likely (OR: 1.37, 95%CI: 1.2–1.56) to quit tobacco.

#### Pre-existing co-morbidity

Callers who did not have a known co-morbid condition have a 20% lower quitting rate as compared to those with co-morbid conditions (OR: 0.8, 95% CI: 0.71–0.91).

## Discussion

The objective of the study is to present the call attended by NTQLS Mumbai and understand the factors influencing the process of tobacco quitting that are fulfilled by the results. This shows that NTQLS, TMC Mumbai play an important role in tobacco control and help in identifying the factors that serve as determinants for tobacco quitting. The findings reflect that in the years 2021–2022, a total of 448,893 calls hit the system. Of these, 127,163 (28.3 %) calls were attended. Of the attended calls, a quit date was set for 21,504 calls (16.9%); of these, 8,276 (38.5%) callers quit tobacco. This shows that people are eager to utilise the services considering we have received over 0.45 million calls in the year 2021–2022 of which only around 30% of the calls could be attended. Of the attended calls, 1 out of 6 people agreed to take counselling and of the people agreeing to the counselling, 1 in 3 people quit tobacco [21]. This reflects that calls hitting the system are high but there is limited infrastructure and manpower to cater to the increasing demand of the population. To meet the increased demand for quit line services there is a need to strengthen the workforce at quit line centres as well as the services provided. Additionally, to expand the tobacco cessation services, an integrative approach can be employed through the linkage of the NTQLS and the tobacco cessation clinics under the National Tobacco Control Programme at the district level which aims to facilitate free pharmacotherapy and counselling services for tobacco cessation [7]. The study results show the odds of factors influencing tobacco quitting. Individuals with previous quit attempts, who never consumed alcohol, consumed tobacco within 6–30 minutes and 30–60 minutes after waking up had higher quitting rates. While, female callers, private sector workers, individuals consuming more than ten tobacco units/packets, tobacco used for more than 10 years, expenditure of more than 5,000 rupees on tobacco and those with no known co-morbid conditions were less likely to quit tobacco.

Female callers show a lesser tendency to quit tobacco as compared to male callers. As per our experience from NTQLS, Mumbai, this can be attributed to the low awareness among females regarding quitline services, social stigma, busy schedules and lack of dedicated personal time. Our study results for gender variable were in accordance with the study conducted using GATS India (2009–10) [15] where a significant association was found with male gender and GATS 2016 Ethiopia data [18] stating that women were less successful in quitting tobacco. However, a study in Andhra Pradesh found contrasting results with women having higher quitting than males [16].

A study to assess caller characteristics based on the data from Korean Tobacco Cessation Service found that women prefer cessation services that are web or telephone-based to ensure more privacy [25]. A study by Li and Okamoto [26] highlighted social stigma as a main barrier for women to access these services; as women are mostly reluctant to disclose their habit of tobacco consumption therefore facing difficulty in tobacco quitting. A study conducted in the Netherlands found the main barrier for women in quitting tobacco is psychological as it involves emotion and stress as compared to environmental factors seen in men such as second-hand smoke and peer pressure. Another explanation that the study highlighted was women’s physical nicotine dependency. Physical nicotine dependency was lower in women than men but women tend to have higher behavioural and mental nicotine dependency [27]. Studies have also stated hormonal changes throughout the menstrual cycle have an impact on tobacco quitting in women [28]. Women also tend to metabolize nicotine faster than men; therefore, the standard dose of nicotine replacement therapy does not work as effectively in women as it does in men [27].

In the present study, drivers/farmers/labourers have a high quitting rate as compared to private sector callers. It can be due to work-related stress experienced by the private sector workers making it difficult for them to quit tobacco. A study has observed that among corporate sector workers, their exposure to second-hand smoke and peer pressure are the major reasons that make tobacco quitting difficult among private sector workers [29].

For tobacco consumption habits and tobacco dependency, it is seen that callers who consumed tobacco for less than 10 years are more willing to quit as compared to those who consumed it for more than 10 years. Data shows that callers who use tobacco within 30–60 minutes of waking up have a high quit rate as compared to individuals who use tobacco less than 5 minutes after waking up which indicates that individuals having a high dependency on tobacco have a lesser quitting rate. These results are as per the study conducted using GATS India 2016 data [18], and studies conducted in Qatar [11] and China [30].

In the present study, the callers who consume up to ten tobacco units/packets consumption per day have a higher rate of quitting than those who consume more than ten units/packets of tobacco. This finding is in line with the findings of the studies by Li *et al* [31] and Myung *et al* [32]. Both these studies highlight that fewer units of tobacco consumption per day indicate that the smoker is minimally dependent on nicotine, thus making it easier to quit [31–33].

Individuals attempting to quit tobacco for the first time have almost 50% higher quit rates than those who have tried to quit before. These results pertaining to tobacco dependency are contradictory to the study conducted in the smoking clinic of a northeastern Malaysian public hospital [33] and similar to the study in Qatar [11]. It was found that those who previously made a tobacco quitting attempt and could not accomplish it perceived themselves as a failure. This also impacted their future quitting intentions. On the other hand, first-time quitters are highly driven and motivated which helps them quit tobacco easily as compared to their counterparts [11].

Our study shows those people who are spending less than Indian Rupees INR 1,000 (~12 USD) have a higher quit rate than those who are spending more than INR. 1,000 (~12 USD). Higher expenditure was shown to higher dependency on tobacco, thus making it difficult to quit tobacco. Saving money was also listed as one of the reasons to quit tobacco [34].

Individuals who have no other addiction like alcohol with tobacco have almost 40% higher quitting rate than those who are consuming alcohol as well. Callers having co-morbid disorder have around 20% higher quitting rate as compared to those with no co-morbid disorder. Those having other addictions to tobacco, such as alcohol were more likely to face adverse health effects due to tobacco. This was aggravated by the presence of co-morbidities. Such individuals perceive health as a major priority thus helping them to quit tobacco as compared to their counterparts who perceive smoking as a mere threat to their health [33].

To summarize, it was observed that the caller’s gender, occupation, number of tobacco units/packets used, tobacco dependency, expenditure on tobacco, previous quit attempts, alcohol/substance use and co-morbidities are the factors that influence tobacco quitting. Reflecting on the findings of the study, the NTQLS, TMC Mumbai centre must emphasize the factors significant to females and provide additional stress-relieving advice to private sector callers. A rigorous and tailor-made counselling plan with additional efforts should be undertaken to counsel callers with high tobacco dependency, those with high expenditure on tobacco, those consuming alcohol with tobacco and those with pre-existing co-morbid conditions.

Based on the aforementioned finding, NTQLS, Mumbai provides the following recommendation;

1. These variables should be considered during call assessment by the counsellors, where those with callers’ characteristics being predictive of quitting tobacco easily shall be identified and targeted by the counsellor.
2. Similarly, a different counselling strategy with an increased dedicated counselling time should be provided to those callers with characteristics not predictive of tobacco quitting.
3. The counsellors can also help the caller to connect with the nearest government district centre that provides necessary medical help in tobacco cessation.
4. In addition to this, with the consent of the caller his/her family member or next of kin whom the caller trusts; can also be counselled about the necessary support provided to the caller during his/her tobacco-quitting journey. This helps in enhancing social support and making the environment helpful to quit tobacco [35].

NTQLS is a freely available, easy-to-access and convenient service that individuals willing to quit tobacco can utilise without any barrier to the physical location of the individual. This also helps in avoiding the stigma that individuals face when attempting to quit tobacco. Importantly, for cessation counselling one of the barriers encountered was that pertaining to language; thus, impeding the adherence of the individuals to tobacco cessation [36, 37] however NTQLS provides counselling to the individual in the local language or the language with which the individual is comfortable.

Tobacco-related risk factors are highly prevalent in LMICs [38]. Being one of the leading risk factors of NCDs worldwide contributing to premature mortality, tobacco consumption directly impedes the progress of the SDGs (SDG 3.4) [39]. The Southeast Asia region accounts for 81% of the world’s smokeless tobacco users and 1/3rd of the world’s school children 13–15 years using tobacco are from this region [40].

To tackle this region-specific burden of tobacco, the Dili Declaration on Tobacco Control was adopted at the 68th Session of the South-East Asia Regional Committee. To contain the growing tobacco epidemic, a political commitment was made to develop, strengthen, implement and accelerate evidence-based tobacco control policies at the country level. It is specially focused on challenges posed by smokeless tobacco and electronic nicotine delivery systems [40].

Cessation services are crucial to alleviate tobacco use and quitline serves as a standard for tobacco cessation [41]. In 2018, tobacco quitline services were available in 66 countries [42]; however, the Association of Southeast Asian Nations countries still have limited facilities that provide tobacco cessation support [43]. Considering the need to strengthen tobacco cessation services in the Southeast Asia region [44], NTQLS, TMC Mumbai India can serve as a training hub in providing support to strengthen the cessation services in the region.

## Strengths and limitations

The study highlights the factors influencing tobacco quitting, incorporating the sociodemographic characteristics as well as variables of tobacco consumption habits along with details of substance use, alcohol consumption and existing co-morbidities. There is a need for such services

The limitation of the study is that the study is based on a selected sample of people who quit tobacco through NTQLS, TMC Mumbai. The callers are confirmed as quitters on a telephonic conversation with the counsellor; therefore, there is a possibility of reporting bias.

## Conclusion

The study highlights the predictors of tobacco quitting. These predictors will be emphasized and considered while planning the counselling strategy for the callers. This will improve the quality of the tailor-made, caller-specific counselling strategy used to help the caller quit tobacco. We observed that there is an increase in the demand for quitline services. Thus, the quitline service and its workforce must be strengthened. These findings will help in developing interventions that will yield better outcomes. A strong foundation of counselling and additional tailored interventions focusing on significant predictors can bring the end goal of NTQLS of tobacco control to fruition.

## List of abbreviations

CCE, Centre for Cancer Epidemiology; GATS, Global Adult Tobacco Survey; LMIC, Low-and middle-income countries; MoHFW, Ministry of Health and Family Welfare; NCD, Noncommunicable disease; NTQLS, National Tobacco Quitline Services; SDG, Sustainable Development Goals; TMC, Tata Memorial Centre; VPCI, Vallabhbhai Patel Chest Institute; WHO, World Health Organisation.

## Conflicts of interest

No potential conflict of interest was reported by the author(s).

## Statement of ethical compliance

This was a service-based project where verbal informed consent was taken through telephone from the caller. Caller-related information collected during routine calls was de-identified during the data analysis and manuscript preparation; therefore, ethical approval for this manuscript was not required.

## Author contributions

AB: conceptualisation, methodology, writing, editing, critical reviewing, overall supervision; SM: Writing, editing, data management, literature review; SB: Writing, editing, data management; PC: Writing, editing, data management; GO: counselling and data collection; RS: counselling and data collection; MY: counselling and data collection; SS: quality control of counselling, data quality assurance, data management; SG: quality control of counselling, data quality assurance, data management; PC: critical review of the manuscript, technical guidance, overall supervision.

## Figures and Tables

**Table 1.** Age distribution of the calls for which the quit date was set.

Age group	Frequency	Percentage (%)
Less than 18	349	1.6
18–24	7,438	34.6
25–34	8,454	39.3
35–44	3,177	14.8
45–54	1,229	5.7
55–64	503	2.3
65 above	217	1.0
No information	137	0.6
Total	21,504	

**Table 2.** Percentage distribution of quitters and non-quitters for year 2021–2022 at NTQL centre, TMC Mumbai.

Percent distribution of quitters and non-quitters				
Characteristics	^#^Quitters		^*^Non-quitters	
	Number (8.276)	% (30.4)	Number (18.917)	% (69.6)
Age-group				
<35	6,258	75.6	14,278	75.5
35–44	1,178	14.2	2,823	14.9
Above 45	811	9.8	1,670	8.8
Unknown	29	0.4	146	0.8
Caller gender				
Male	8,180	98.9	18,500	97.8
Female	96	1.1	417	2.2
Occupation				
Driver/farmer/Labour	2,894	35.0	5,847	30.9
Private sector	1,713	20.7	5,392	28.5
Student	1,009	12.2	2,394	12.7
Others	1,298	15.7	3,171	16.8
Unknown	1,362	16.5	2,113	11.2
Income (INR)				
Below 30,000	6,267	75.7	13,069	69.1
Above 30,000	911	11.0	2,642	14.0
Unknown	1,098	13.3	3,206	16.9
Education				
Primary	1,102	13.3	2,613	13.8
Secondary	3,441	41.6	7,003	37.0
Diploma & above	3,591	43.4	8,711	46.0
Unknown	142	1.72	590	3.1
Marital status				
Married	4,362	52.7	10,003	52.9
Unmarried	3,845	46.5	8,592	45.4
Others	22	0.3	77	0.4
Unknown	47	0.6	245	1.3
Tobacco type				
Smokeless	6,688	80.8	14,163	74.9
Smoking	1,005	12.1	3,110	16.4
Both	583	7.0	1,644	8.7
Number of years tobacco use				
Up to 10 years	3,512	42.4	7,647	40.4
Above 10 years	1,970	23.8	5,052	26.7
Unknown	2,794	33.8	6,218	32.9
How soon after waking do you use tobacco				
Within 5 minutes	4,424	53.5	10,857	57.4
6–30 minutes	1,625	19.6	3,257	17.2
30–60 minutes	1,170	14.1	2,406	12.7
> 60 minutes	1,016	12.3	2,169	11.5
Unknown	41	0.5	228	1.2
Units (Smoke)/Packets (Smokeless) of tobacco used (per day)				
Less than 10	2,542	30.7	5,138	27.2
Greater than 10	3,614	43.7	8,857	46.8
Unknown	2,120	25.6	4,922	26.0
Previous quit attempt				
Yes	6,145	74.3	14,975	79.2
No	1,092	13.2	2,076	11.0
Unknown	1,039	12.6	1,866	9.9
Expenses on tobacco (per month)				
Less than 1,000	4,840	58.5	10,303	54.5
1,000–5,000	2,899	35.0	6,629	35.0
>5,000	312	3.8	1,290	6.8
Unknown	225	2.7	695	3.7
Alcohol taking status				
Yes	1,669	20.2	5,219	27.6
No	6,416	77.5	13,261	70.1
Unknown	191	2.31	437	2.3
Other substance use				
Yes	1,719	20.8	2,939	15.5
No	6,336	76.6	15,388	81.3
Unknown	221	2.7	590	3.1
Comorbidity				
Yes	2,585	31.2	5,597	29.6
No	4,879	59.0	12,514	66.2
Unknown	812	9.8	806	4.3

# Quitters are the number of tobacco users that quit tobacco in the years 2021–2022

* Non Quitters include the tobacco users who could not quit tobacco or relapsed in the year 2021–2022 irrespective of the timeframe in which the quit date was set. For example; non-quitters also include callers for whom the quit date was set in 2020 but found to be relapsed or could not quit during follow-up calls made in the years 2021–2022

**Table 3.** Multiple logistic regression model identifying the factors influencing tobacco quitting among callers at NTQLS, Mumbai for the years 2021–2022.

Predictors	Univariate logistic regression		Multiple logistic regression	
	U.A. Odds ratio (95% CI)	*p*-value	Adj. Odds ratio (95% CI)	*p*-value
Age-group (In years)				
Less than 35	1		1.00	
35–44	0.95 (0.88–1.03)	0.19	0.95 (0.81–1.1)	0.50
Above 45	1.11 (1.01–1.21)	0.02	0.99 (0.84–1.19)	0.95
Caller gender				
Male caller	1.00		1.00	
Female caller	0.67 (0.55–0.82)	0.00	0.59 (0.35–0.99)	0.05
Education				
Diploma and above	1.00		1.00	
Primary	1.02 (0.94–1.11)	0.58	0.94 (0.79–1.11)	0.46
Secondary	1.19 (1.13–1.26)	0.00	1.05 (0.92–1.19)	0.50
Marital status				
Unmarried	1.00		-	
Married	0.97 (0.92–1.03)	0.33	-	-
Others	0.64 (0.40–1.03)	0.06	-	-
Occupation				
Driver/farmer/Labour	1.00		1.00	
Private sector	0.64 (0.60–0.69)	0.00	0.7 (0.61–0.81)	0.00
Student	0.85 (0.78–0.93)	0.00	1.05 (0.8–1.39)	0.72
Others	0.83 (0.76–0.89)	0.00	1.01 (0.85–1.19)	0.93
Income				
Above 30,000	1.00		1.00	
Below 30,000	1.39 (1.28–1.51)	0.00	1.11 (0.93–1.32)	0.24
Tobacco type				
Smokeless	1.00		1.00	
Smoking	1.46 (1.35–1.58)	0.00	1 (0.84–1.21)	0.96
Smoking and smokeless both	1.1 (0.98–1.24)	0.12	0.97 (0.78–1.22)	0.82
No. of years tobacco use				
Upto 10 years	1		1.00	
Above 10 years	0.85 (0.80–0.91)	0.00	0.85 (0.73–0.97)	0.02
How soon after waking do you use tobacco				
Less than 5 minutes	1.00		1.00	
6–30 minutes	1.22 (1.14–1.31)	0.00	1.29 (1.12–1.49)	0.00
30–60 minutes	1.19 (1.10–1.29)	0.00	1.26 (1.05–1.51)	0.01
>60 minutes	1.15 (1.06–1.25)	0.00	1.13 (0.91–1.40)	0.27
Units (Smoke)/ Packets (Smokeless) of tobacco used (per day)				
Less than 10	1.00		1.00	
Greater than 10	0.82 (0.78–0.88)	0.00	0.7 (0.61–0.79)	0.00
Previous quit attempt				
Yes	1.00		1.00	
No	1.28 (1.18–1.39)	0.00	1.48 (1.25–1.75)	0.00
Expenses on tobacco per month (INR)				
Less than 1,000	1.00		1.00	
1,000–5,000	0.93 (0.88–0.98)	0.01	0.84 (0.74–0.95)	0.01
More than 5,000	0.51 (0.45–0.59)	0.00	0.58 (0.44–0.77)	0.00
Alcohol taking status				
Yes	1.00		1.00	
No	1.51 (1.42–1.61)	0.00	1.37 (1.2–1.56)	0.00
Other substance use				
Yes	1.00		1.00	
No	0.7 (0.66–0.75)	0.00	0.9 (0.77–1.05)	0.18
Comorbidity status				
Yes	1.00		1.00	
No	0.84 (0.8–0.89)	0.00	0.8 (0.71–0.91)	0.00

U.A., Unadjusted; Adj., Adjusted; CI, Confidence interval
